# The influence of timing of Maternal administration of Antibiotics during cesarean section on the intestinal Microbial colonization in Infants (MAMI-trial): study protocol for a randomised controlled trial

**DOI:** 10.1186/s13063-019-3552-8

**Published:** 2019-08-05

**Authors:** Thomas H. Dierikx, Daniel J. C. Berkhout, Laura Visser, Marc A. Benninga, Guus Roeselers, Nanne K. H. de Boer, Johanna I. P. de Vries, Tim G. J. de Meij

**Affiliations:** 1 Department of Paediatric Gastroenterology, Amsterdam UMC, location VUmc, 1081 HV Amsterdam, The Netherlands; 2 Department of Gynaecology and Obstetrics, Amsterdam UMC, location VUmc, 1081 HV Amsterdam, The Netherlands; 3 Department of Paediatric Gastroenterology, Amsterdam UMC, location AMC, 1105 AZ Amsterdam, The Netherlands; 4 Danone Nutricia Research, 3584 CT Utrecht, Netherlands; 5 Department of Gastroenterology and Hepatology, Amsterdam UMC, location VUmc, AG&M Research Institute, 1081 HV Amsterdam, The Netherlands

**Keywords:** Caesarean section, Antibiotics, Intestinal microbiota, Infants

## Abstract

**Background:**

A disturbance in the early colonisation of the gut by microorganisms is associated with an aberrant innate immune system and a variety of clinical conditions later in life. Several factors are considered to influence this initial colonisation, including maternally administered antibiotics during pregnancy and delivery. Recent revisions to international obstetric guidelines have resulted in the exposure of all infants born by caesarean section (CS) to broad-spectrum antibiotics perinatally. To date, the consequences of these new guidelines on neonatal gut colonisation and the associated short- and long-term health implications have not yet been addressed. The aim of this study is to investigate the influence of the timing of antibiotic administration during CS to the mother on the course of neonatal intestinal colonisation up to 2 years of age.

**Methods/design:**

This single-centre randomised controlled trial will recruit 40 women scheduled for an elective CS. The subjects will be randomised to receive 1500 mg of cefuroxime intravenously either prior to the skin incision (*n* = 20) or after clamping of the umbilical cord (*n* = 20). Levels of cefuroxime in cord blood will be determined for exposed neonates. Faecal samples from the children will be collected on days 1, 7 and 28 days and at 2 years old and analysed by 16S sequencing. Shannon-diversity indices, absolute and relative abundances, and unsupervised and supervised classification methods will be used to evaluate the effect of the timing of intrapartum cefuroxime administration on the composition of the microbiota. The outcomes for both study groups will be compared to the intestinal microbiota of vaginally born infants (*n* = 20). To detect possible effects on health state, a questionnaire on health-related issues will be taken at the age of 2 years.

**Discussion:**

In the proposed study, changes in the intestinal microbiota of 40 children born by CS will be followed until the age of 2 years. Research on this topic is necessary since significant effects relating to the timing of antibiotic administration on microbial colonisation may conflict with the current guidelines, as this may have health consequences later in life.

**Trial registration:**

Netherlands Clinical Trial Registry, NTR6000. Retrospectively registered on 25 July 2016.

**Electronic supplementary material:**

The online version of this article (10.1186/s13063-019-3552-8) contains supplementary material, which is available to authorized users.

## Background

The human gut harbours an immense number of microbial cells, estimated to be 10^13^–10^14^ microorganisms [1]. The intestinal microbiota has an essential role in maintaining overall health through several different mechanisms, including protection against pathogens, priming of the immune system, digestion of food and synthesis of essential vitamins [2–4]. In contrast, disturbances of the composition of the intestinal microbiota, especially early in life, are associated with the development of numerous diseases and clinical conditions such as asthma, allergies, inflammatory bowel disease, atopy, necrotising enterocolitis and obesity [2, 5–11]. Despite increasing evidence for this crucial role of the intestinal microbiota in health and disease, little information exists on colonisation early in life and on environmental factors that may affect its composition. Information on microbial dynamics during the neonatal period is pivotal, since early microbial colonisation is considered to be crucial for the microbial composition later in life.

It is generally considered that the foetal gut is sterile and that its colonisation is initiated by exposure to bacteria at birth. However, recent studies have demonstrated that gut colonisation begins in utero [12]. In the first days following birth, environmental factors like (breast)feeding, skin contact and medication further contribute to colonisation [13]. The influence of the route of delivery and the use of antibiotics during a caesarean section (CS) on neonatal microbial colonisation has not been completely clarified. Neonates born by CS have delayed intestinal colonisation compared to vaginally delivered children, since there is no contact with the maternal vaginal and faecal microbiota and the perineal skin [14–16]. In addition, infants born by CS seem to have a decreased microbial diversity, possibly persisting for a prolonged period of at least 2 years [15]. Consequently, these infants are considered to have an increased risk of developing diseases associated with early aberrations in gut microbiota [15, 16]. However, studies on the role of the route of delivery on neonatal colonisation have few participants and short follow-up periods, while the reported results are inconsistent. In addition, there is limited information on the impact of the timing of maternal antibiotic administration during a CS on neonatal colonisation.

According to the guidelines of the National Institute for Health and Care Excellence (NICE) in the United Kingdom [17], prophylactic antibiotics should be given to all women who are undergoing a CS prior to the skin incision. However, the previous guidelines advised administration of antibiotics after clamping of the umbilical cord. This adjusment has been shown to decrease the risk of total infectious morbidities, without an increase in adverse neonatal outcomes. Maternal infectious morbidity affects around 5–10% of women undergoing CS [17, 18]. In a recent meta-analysis, infectious morbidity affected 3.9% of women receiving antibiotics preoperatively, compared to 6.9% of women who received antibiotics after clamping of the umbilical cord (relative risk 0.57). This reduction was predominantly caused by a reduction of endometritis and fewer wound infections [18]. Obviously, when antibiotics are administered prior to skin incision, the infant will be exposed too, whereas infants are not exposed if the antibiotic is administered after clamping of the cord. The effects of this protocol adjustment on neonatal outcomes were exclusively evaluated in the short term, including the effects on suspected sepsis that required a workup [18, 19]. The long-term consequences of the timing and type of antibiotics, such as the influence on neonatal intestinal colonisation, priming of the immune system and on the incidence of auto-immune diseases, like coeliac disease, inflammatory bowel disease, asthma and allergies, have not yet been addressed [20].

## Methods/design

### Study aim

As mentioned previously, infants born by CS have delayed intestinal colonisation and decreased microbial diversity. We hypothesise that exposing these caesarean-delivered infants to intrapartum-administered broad-spectrum antibiotics would further influence the colonisation process. Therefore, the aim of the present study is to evaluate the effects of the timing of the maternal administration of antibiotics during CS on the early neonatal intestinal microbiota and the microbiome until the children are 2 years old.

### Objectives

Thus, the primary objective of this study is to describe the microbiome of the intestinal colonisation in the first month of life and at 2 years of age in two groups of children born by CS: (1) infants exposed to maternally administered antibiotics prior to CS (current NICE guideline) and (2) unexposed neonates from mothers receiving antibiotics after clamping of the cord (former NICE guideline). These outcomes will be compared with each other and to a third study group of vaginally born infants.

Secondary objectives are to investigate whether there are differences at the age of 2 years regarding the health state of children born by CS, in terms of the timing of antibiotic administration. Moreover, we plan to evaluate the association between maternal vaginal and rectal microbiota composition and neonatal gut colonisation in infants born vaginally and by CS. For CS, transmission will be compared between infants born according to antibiotic exposure following the previous and the current guidelines.

### Study design and setting

**Fig. 1:**
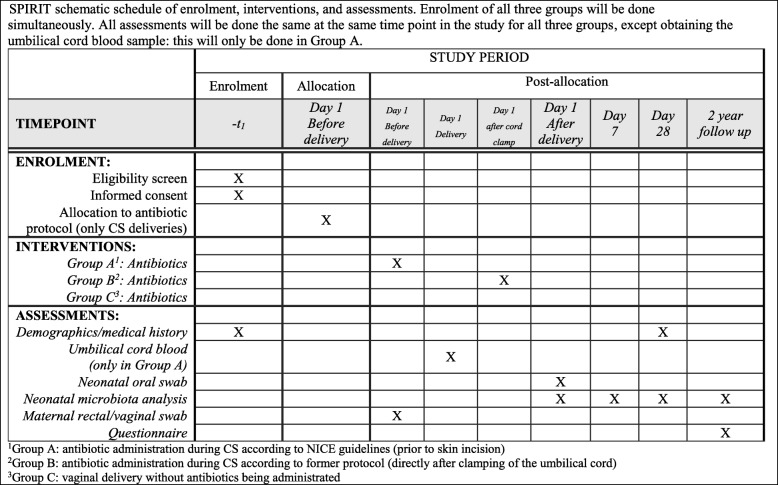
SPIRIT schematic schedule of enrolment, interventions, and assessments. Enrolment of all three groups will be done simultaneously. All assessments will be done the same at the same time point in the study for all three groups, except the umbilical cord blood sample, which will be collected for group A only

**Fig. 2:**
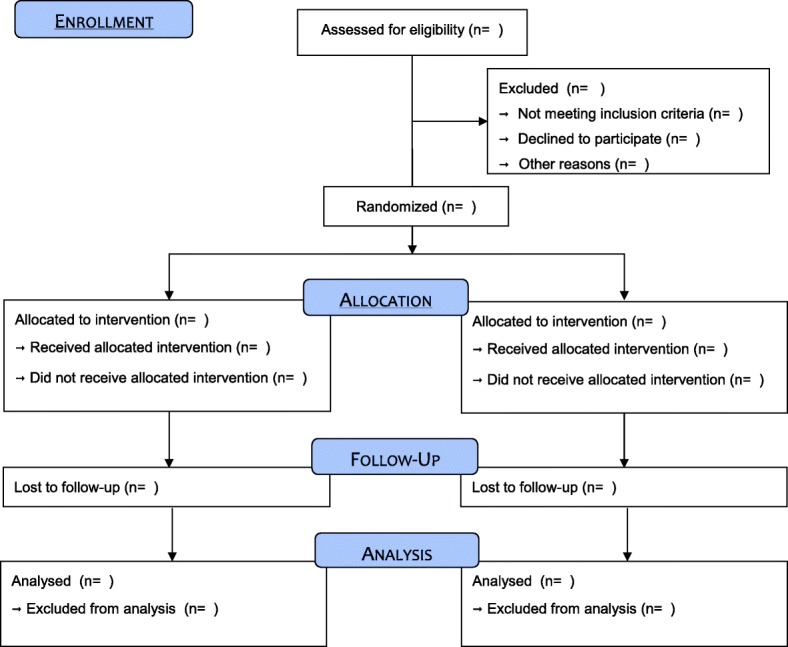
Flow diagram of study participants

This is a single-centre randomised-controlled trial. It will be conducted at the Department of Obstetrics and Gynaecology and the Paediatric Department of the Medical Centre of Vrije University (VUmc), which is part of the University Medical Centre Amsterdam. The protocol was designed in accordance with the SPIRIT statement [21] (Additional file 1). The schedule of enrolments, allocation and assessments is given in Fig. 1. A flow diagram of the progress through the planned study phases is shown in Fig. 2.

### Patient and public involvement

Patients and the public were not involved in the design of the study.

### Subjects and study population

The study population consists of three groups. The first two groups include 40 pregnant women visiting the outpatient clinic of the Department of Obstetrics and Gynaecology at VUmc during the last trimester of pregnancy. They must have an uncomplicated pregnancy so far and be scheduled for CS. An uncomplicated pregnancy is defined as a normotensive singleton pregnancy, with a normal-weight foetus without maternal or foetal risk factors, delivered without complications at a gestational age of more than 37 weeks. Twenty women will be treated according to the current NICE guidelines for antibiotic administration during CS (group A). These subjects will receive 1500 mg cefuroxime intravenous 30 min prior to skin incision. The remaining 20 women will be treated according to the former protocol (group B) and will be administered 1500 mg cefuroxime intravenous directly after clamping of the umbilical cord. Allocation to a group will be randomised in permuted blocks of 10 using www.random.org. Allocation to a group will be blinded only for the statistician performing the analysis. The third group will include 20 pregnant women with an uncomplicated pregnancy as described above, admitted for outpatient vaginal delivery in VUmc (group C). Exclusion criteria for this group include antibiotics administration during pregnancy or labour and premature rupture of membranes (Table 1). The women delivering vaginally will be recruited at the same time as the women in the other two groups. The neonates born at ≥37 weeks gestational age from mothers included in one of the three study groups will be included following informed consent from both their parents.

**Table 1:** Maternal and neonatal exclusion criteria

Maternal exclusion criteria	
Delivery < 37 weeks gestation	
Aged ≤17 years	
Body mass index ≥ 25	
Antibiotic use during pregnancy	
Antibiotic use during labour (group C)	
Immunosuppressive use within 3 months prior to delivery	
Inflammatory bowel disease	
Coeliac disease	
Rupture of membranes before CS (groups A and B)	
Rupture of membranes > 18 h (group C)	
Diabetes mellitus type I or II	
Gestational diabetes requiring insulin	
History of gastro-intestinal surgery	
Alcohol or tobacco use in second or third trimester	
Use of recreational drugs during pregnancy	
Maternal antibiotic use during first month of neonatal life	
Neonatal exclusion criteria	
Congenital gastro-intestinal anomalies	
Gastro-intestinal surgery during first month of life	
Antibiotic or immunosuppressive medication use during first month of life	

Standard demographic and obstetric variables will be collected. Other variables, including nutrition of the neonate (breast milk or formula) during the first month of life, birthweight, Apgar scores after 1 and 5 min and presence of meconium-stained amniotic fluid, will be collected. Data on maternal and neonatal medication use during pregnancy and the first month postnatally will be collected, as well as data on maternal weight, neonatal length and maternal diet (vegetarian or non-vegetarian).

### Exclusion criteria

An overview of the maternal and neonatal exclusion criteria is provided in Table 1. The criteria are predominantly clinical variables considered to influence the composition of the gut microbiota.

### Sample size calculation

A formal power analysis could not be performed as there are currently no relevant data in the literature on the 16S rRNA gene-sequencing technique. For this study, we aim to include 60 children: 20 born vaginally and 40 born by CS (20 from mothers receiving antibiotics before CS and 20 after clamping of the umbilical cord).

### Study materials and samples

At an outpatient clinic prior to their scheduled CS or vaginal delivery, all women will be asked to take a vaginal swab and a rectal swab. The swabs will be stored at − 20 °C directly after sampling.

To determine to what extent neonates from group A have been exposed to cefuroxime, we will collect umbilical cord blood from these infants after clamping of the cord and delivery of the placenta. The blood will be collected in an ethylene-diamine-tetra-acetic acid (EDTA) tube. Directly after collection, the samples will be stored on ice before further handling.

Parents are asked to collect faecal samples from the neonate (approximately 2 g or more), in sterile stool containers (10 mL, Frickenhausen, Germany) on days 1, 7 and 28 and at age 2 years (at home). These samples are stored at − 20 °C (at home in a domestic freezer), within 1 hour of collection.

An oral swab will be taken from the neonate directly after birth to evaluate the presence of maternal microorganisms (vaginal and faecal). The swabs are stored at − 20 °C within 1 hour following collection.

When the child reaches the age of 2 years, a questionnaire will be sent to the parents focusing on the child’s health status. Items include information on allergies, use of medication, hospital admissions, number of visits to the general practitioner and the use of antibiotics between birth and collection of the follow-up sample.

Depending on the results of the analyses, we will schedule a follow-up of the children later in early childhood.

### Data management and monitoring

All study participants will receive a study identification number. Electronic data will be kept in a secure password-protected database. The data collected will be stored at the research unit in the study centre. All data will be securely stored, backed up and retained for 15 years. Additional consent from both parents will be obtained to store and use this data in future studies. Only relevant members of the research team will have access to the data. A data safety monitoring committee is not required according to the guidelines proposed by Ellenburg et al. [22]*.* The study will be independently monitored at study close-out. No interim analyses will be performed.

### DNA extraction and microbiota analysis using 16S rRNA gene sequencing

Stool samples from infants will be collected, as reported above, on days 1, 7 and 28 and at 2 years old. DNA will be extracted, as documented previously by Daniels et al. [23]. From the purified faecal DNA, extracts of the V3-V4 hypervariable regions of the bacterial 16S rRNA gene will be amplified, using universal primers S-D-Bact-0341-b-S-17 (forward 5′-CCTACGGGNGGCWGCAG-3′) and S-D-Bact-0785-a-A-21 (reverse 5′-GACTACHVGGGTATCTAATCC-3′) [24]. Sequencing will be performed by LifeSequencing SL (Valencia, Spain) on an Illumina MiSeq instrument (San Diego, California, USA).

### Statistical analysis

Baseline characteristics will be summarised for all subjects by treatment regimen and compared between groups.

The primary outcome will be differences in the microbiota between the two groups of infants born by CS on day 28. An adaptation of QIIME v1.9.0 [25] will be used to analyse the sequenced data. Briefly, sequences will be clustered into operational taxonomic units based on 97% sequence identity as a proxy for bacterial species using VSEARCH v2.03 against the RDP gold database and by excluding chimeric sequences [26]. Taxonomic assignment will be performed using the RDP classifier [26] against the SILVA119 database [27]. The species diversity (α-diversity) of faecal and salivary samples will be calculated using Faith’s phylogenetic diversity [28] and the Shannon index for diversity [29] and by applying a correction for differences in sequencing depths by rarefaction. Furthermore, absolute and relative abundances as well as unsupervised and supervised classification methods will be used to evaluate the influence of timing of intrapartum cefuroxime administration on the composition of the microbiota.

Clinical outcomes at the 2-year follow-up will be compared using Student’s *t*-test or a Mann–Whitney *U* test for continuous variables depending on whether the variables are distributed normally. A χ^2^ test or Fisher’s exact test will be used to compare normal and non-normal dichotomous variables, respectively. Differences between groups will be presented for continuous outcomes as differences in means or differences in medians (for normal or non-normal distributions, respectively) along with a 95% confidence interval. For dichotomous outcomes, the relative risk and number needed to treat will be determined along with a 95% confidence interval. All comparisons will be made between the randomised exposed (group A) and the unexposed (group B) infants born by CS. Furthermore, all analyses will compare infants from both CS groups together (groups A and B) with children born vaginally (group C) and lastly infants born vaginally will be compared with the two CS groups separately. Differences between study groups will be considered significant if *p* < 0.05.

If differences exist between the groups in terms of feeding strategies or other baseline characteristics, we will adjust for this in the statistical analysis. Since nutrition affects the microbiota and antibiotics might be present in a limited concentration in breast milk, we will perform the same analysis for infants fed only by formula and for infants breastfed only and adjust for this factor. Furthermore, the concentration of cefuroxime in the umbilical cord blood will be valuable in interpreting the influence of the these factors.

## Discussion

Recent revisions to international obstetric guidelines have resulted in the exposure of all infants born by CS to antibiotics, which possibly affects their microbiome. This could hypothetically increase the risk of the clinical conditions mentioned above. The number of infants born by CS has increased unprecedentedly in recent years, currently reaching a rate of over 27% in developed countries. In some countries, it even exceeds 50% [30]. Insights into the possible effects on the intestinal microbiota of early antibiotic exposure and the role of the intestinal microbiota in health and disease later in life are, therefore, essential to ensuring the safety of the current guidelines for infants. However, few studies have addressed this topic.

Existing studies investigating the effect of antibiotics administered during labour on neonatal gut colonisation have merely focused on the effect of penicillin prophylaxis for women colonised by group B *Streptococcus*. These studies demonstrated that vaginally born infants exposed to penicillin have a less diverse microbiota compared to unexposed infants [31–34].

There are few studies on the impact of cephalosporin administration during CS. One study showed increased Firmicutes abundances and an almost entire depletion of Bacteroidetes species at the postnatal age of 3 months. In contrast, at the age of 1 year, they did not observe any differences in the microbiota of infants born by elective CS [35]. However, early microbial dysbiosis at the age of 3 months could already have affected the development of the innate and adaptive immune systems, resulting in long-lasting pathologic consequences that persist even after normalisation of the composition of the gut microbiota [36]. A second study demonstrated a delayed colonisation with Actinobacteria, a concurrent lack of Bacteroidetes and a higher Firmicutes abundance compared to unexposed vaginal deliveries [34]. However, it should be acknowledged that both studies compared antibiotic-exposed infants to vaginally born unexposed infants. Since the route of delivery has an impact on the microbial composition [15, 37, 38], both studies lack the ability to draw firm conclusions about the influence of antibiotic administration during CS on the neonatal microbiota.

### Strengths

The main strength of this study is the applied randomised controlled design. Another important strength is the determination of cefuroxime levels from umbilical cord blood, which will provide detailed information about the degree of antibiotic exposure for the infant. Another strength is the 16S sequencing technique, which allows for microbiota profiling at the species level [24, 39].

### Limitations

There are also some limitations. Because our study will include patients only from a single tertiary hospital, our results cannot automatically be extrapolated to a more general population. Secondly, only a limited number of children (20 born vaginally and 40 born with CS) will be included. The number of anticipated participants is rather low but will allow us to determine whether there are significant differences in neonatal colonisation, which would hypothetically challenge the safety of the NICE guidelines for infant health. Moreover, we aim to investigate whether there are differences regarding health state, since a disturbance in the composition of the intestinal microbiome may have a key role in the pathogenesis of numerous diseases. We scheduled a follow-up at 2 years; however, often the relevant diseases develop much later in life and we intend to perform a follow-up analysis at the age of 5 years.

Furthermore, the microbiota plays a key role in the function and development of the host immune system [40]. In this study, we will not investigate the effect of the timing of antibiotic administration on this immunisation process.

Hypothetically, infants born without being exposed to cefuroxime during CS could still be exposed to antibiotics through lactation. However, cefuroxime has a relatively short half-life in adults and a low peak concentration in breastmilk [40–42]; thus, we consider the potential effect on the composition of the microbiota due to lactation to be negligible. Furthermore, if differences exist between the groups in terms of feeding strategies, we will adjust for this in the statistical analysis.

### Summary

The potential effects of the timing of antibiotic administration during CS on the neonatal microbiome have not yet been fully studied and, thus, remain unclear. In this randomised controlled trial, we aim to provide further insights into the effects on neonatal gut colonisation and the potential health implications for the infant as a result of the new international guidelines. Since disturbances of the colonisation process are associated with a variety of diseases later in life, any observed aberrations in colonisation would hypothetically challenge the safety of these guidelines on infantile health.

### Trial status

Recruitment started in February 2015 following approval from the local ethics committee. Since participants are being recruited from only one tertiary hospital and only women with uncomplicated pregnancies scheduled for a CS are included, the recruitment rate is rather low. Enrolment is expected to be complete by August 2019. Protocol version 3.0 was approved by the regional ethics committee on 29 January 2019.

## Additional file

Additional file 1: SPIRIT 2013 Checklist: Recommended items to address in a clinical trial protocol and related documents. (DOC 121 kb)

## Data Availability

The datasets that will be generated and analysed during the current study are not publicly available but are available from the corresponding author on reasonable request.

## Abbreviations

CS: Caesarean section
NICE: National Institute for Health and Care Excellence
SPIRIT: Standard Protocol Items: Recommendations for Interventional Trials
VUmc: Medical Centre of Vrije University
